# Antibacterial Properties of Calcium Fluoride-Based Composite Materials:* In Vitro* Study

**DOI:** 10.1155/2016/1048320

**Published:** 2016-12-08

**Authors:** Monika Łukomska-Szymańska, Beata Zarzycka, Janina Grzegorczyk, Krzysztof Sokołowski, Konrad Półtorak, Jerzy Sokołowski, Barbara Łapińska

**Affiliations:** ^1^Department of General Dentistry, Medical University of Lodz, Lodz, Poland; ^2^Department of Microbiology and Laboratory Medical Immunology, Medical University of Lodz, Lodz, Poland; ^3^Department of Conservative Dentistry, Medical University of Lodz, Lodz, Poland

## Abstract

The aim of the study was to evaluate antibacterial activity of composite materials modified with calcium fluoride against cariogenic bacteria* S. mutans* and* L. acidophilus*. One commercially available conventional light-curing composite material containing fluoride ions (F2) and two commercially available flowable light-curing composite materials (Flow Art and X-Flow) modified with 1.5, 2.5, and 5.0 wt% anhydrous calcium fluoride addition were used in the study. Composite material samples were incubated in 0.95% NaCl at 35°C for 3 days; then dilution series of* S. mutans* and* L. acidophilus* strains were made from the eluates. Bacteria dilutions were cultivated on media afterwards. Colony-forming unit per 1 mL of solution (CFU/mL) was calculated. Composite materials modified with calcium fluoride highly reduced (*p* < 0.001) bacteria growth compared to commercially available composite materials containing fluoride compounds. The greatest reduction in bacteria growth was observed for composite materials modified with 1.5% wt. CaF_2_. All three tested composite materials showed statistically greater antibacterial activity against* L. acidophilus* than against* S. mutans*.

## 1. Introduction

In dentistry nowadays, as well as in any other branch of medicine, constant development in material science is observed. The ideal restorative material would not only perfectly restore hard dental tissues, but also possess antibacterial properties, as the most common reason for filling removal is secondary caries [1, 2]. Secondary caries is caused by bacterial infection due to microleakage, bacteria presence in dentinal tubules, or poor adhesion of composite material to hard dental tissues [3, 4]. All of the above may lead to pulp infection and cause postoperative complications. Antibacterial properties of restorative materials are of major clinical importance and would allow for less invasive hard dental tissue preparation, highly influencing positive treatment outcome.

In order to reduce microorganisms proliferation on the tooth-resin interface and around dental fillings, various chemical compounds were added to the materials composition: fluoride compounds, chlorhexidine digluconate (CHG), chlorhexidine diacetate (CHA), nanoparticles of amorphous calcium phosphate (NACP), quaternary ammonium dimethacrylate (QADM), 12-methacryloyloxydodecylpyridinium bromide (MDBP), methacryloxylethylcetyl dimethyl ammonium chloride (DMAE-CB), cetylpyridinium chloride (CPC), quaternary ammonium polyethylenimine (PEI), 2-dimethyl-2-dodecyl-1-methacryloxyethyl ammonium iodine (DDMAI), and furanone derivatives [5, 6].

Composite materials due to surface roughness [7–9] and residual monomers released after polymerization [10] favor bacterial colonization much more than other dental materials like amalgam, gold alloys, or glass ionomer cements [11–13]. Bacteria present in biofilm also induce further adhesion of microorganism to composite filling [14, 15]. Also, even correctly applied composite restorations with time undergo hydrolytic degradation due to water sorption, swelling, plasticization, or enzymatic decomposition. The latter may be induced by enzymes, present in saliva or bacteria, as well as by endogenous enzymes present in dentine—metalloproteinases—that cause collagen fibres hydrolysis leading to microleakage [16]. All of the above mentioned result in degradation of filling material, lack of marginal adaptation, and bacterial colonization of enamel, dentine, and cementum leading to restoration replacement and further loss of hard dental tissues [7, 17–23].

Cariostatic effect of fluoride ions is widely known and involves reduction of demineralization, promoting of remineralization, and incorporating fluoride ions in enamel structure as fluoroapatites and hydroxyl-fluoroapatites. Fluoride prevents demineralization process and enhances remineralization of enamel. Both processes take place in low concentration of fluoride ions in enamel amounting up to 0.03 ppm [8, 14, 20, 24–27]. Due to higher organic substance amount in dentine, the fluoride amount indispensable to enhance both processes should be 10 times higher than for enamel [28]. Fluoride release from dental composites extensively limits secondary caries progression [29]. Altogether, fluoride ions by enhancing enamel toughness and increasing its solubility in acidic environment [24, 28, 30–38] control caries process [21].

Kulshrestha et al. [29] performed research on the influence of CaF_2_ nanoparticles (CaF_2_-NPs) on bacteria, both* in vitro* and* in vivo*. The results showed strong antibacterial activity of CaF_2_-NPs against* S. mutans*: almost 90% reduction of biofilm formation, reduced bacteria acid, and exopolysaccharides production. In low pH environment, fluoride ions bind hydrogen ions creating hydrofluoric acid that penetrates bacterial membrane. Hydrofluoric acid inside bacteria dissociates and causes acidification of cytoplasm and inhibits enzymes (enolase and ATPase) [29, 39, 40]. Fluorides in very high concentrations (3040–5700 ppm) cause bacteria cell death [24].

Moreover, fluoride also adversely influences metabolism and adhesion of bacteria cells [25, 26, 30, 31, 41]. Microbes showed decrease of adhesion to biofilm and greater sensitivity to acidic environment in presence of calcium fluoride. The* in vivo* investigation in rats revealed that, after exposure to calcium fluoride nanoparticles,* S. mutans* adhesion to tooth surface decreased. Additionally, CaF_2_ nanoparticles restrain biofilm formation and as a consequence they reduce caries lesions development, due to great fluoride ions release and its influence on bacteria.

Various fluoride salts were added to organic matrix of composite materials (NaF, CaF_2_, KF, SrF_2_, and SnF_2_), yet the fluoride ion release decreased substantially with time, while mechanical properties were deteriorated [36, 38]. The headway in dental material science should focus on developing restorative material that would combine antibacterial and regenerative properties towards hard dental tissues as well as possess optimal mechanical parameters [8].

## 2. Aim of the Study

The purpose was to evaluate antibacterial activity of composite materials Flow Art and X-Flow modified with calcium fluoride against cariogenic bacteria* S. mutans* and* L. acidophilus*.

## 3. Materials and Methods

### 3.1. Sample Preparation

Two flowable composite materials were used in the study: Flow Art (Arkona, Poland) and X-Flow (Dentsply, Germany) and one conventional composite material F2 (Arkona, Poland).

Flow Art is a light-cured composite that consists of dimetacrylic organic matrix (bisphenol A dimethacrylate, dimethacrylate urethane, and triethylene glycol dimethacrylate) containing inorganic fillers (barium-aluminium-silicate glass, pyrogenic silica) and additions (photoinitiator, coinitiator, inhibitor, stabilizers, and pigments). Mineral fillers constitute up to 60% of the composite.

X-Flow is a flowable, light-curing composite that consists of strontium-alumino-sodium fluoro-phosphor-silicate glass, di- and multifunctional acrylate and methacrylate resins, diethylene glycol dimethacrylate (DGDMA), highly dispersed silicon dioxide, UV stabilizer, ethyl-4-dimethylaminobenzoate, camphorquinone, butylated hydroxyl-toluene (BHT), iron pigments, and titanium dioxide. The filler load was 60% in weight and 38% in volume. Filler size (D 50) was 1.6 micrometers [42].

F2 is conventional light-curing composite containing fluoride ions and Bis-GMA, TEGDMA, UDMA, Bis-EMA, and filler (barium-aluminium-borosilicate glass, fluoro-barium-silicate glass, and pyrogenic silica). The filler load is 77% by weight.

Both flowable composite materials were modified with anhydrate powder of calcium fluoride addition (Arcos Organics, Belgium) and 3 study groups were established as presented in Table 1.

F2 composite material, as the one containing fluoride ions, was not modified with CaF_2_ addition.

The samples were prepared using cylindrical silicone molds of 3 mm height and 5 mm in diameter. Composite material was applied into the mold in layering technique and polymerized with halogen polymerizing lamp at light intensity of 800 mW/cm^2^ (Megalux Soft-Start/Mega-PHYSIK Gmbh & Co. KG, Germany). The manufacturer's instructions were as follows:

- Flow Art: 3 mm material layer was polymerized for 30 s; one layer was applied.
- X-Flow: 1.5 mm material layer was polymerized for 20 s; two layers were applied.
- F2: 3 mm material layer was polymerized for 30 s; one layer was applied.

For each study group (3 study groups) and bacteria strain (2 bacteria strains) 15 samples were prepared. The total number of samples equals 90 (3 × 2 × 15 = 90). From each sample two experiments were performed.

### 3.2. Microbank System

Microbiological studies were conducted on two reference strains:* Streptococcus mutans* ATCC 25175 and* Lactobacillus acidophilus* ATCC 4356. The strains were stored in Microbank systems (Biocorp, Poland). The method was based on ceramic balls placed in containers (cryotube). The vial was inoculated with 24-hour pure bacteria culture (McFarland standard 0.5). The bacteria were attached to ceramic balls in cryopreservation media. After 15-minute immersion in cryopreservation media, each ball (with bacteria) was covered with media. Vials can be stored in a freezer in temperatures ranging from −20°C to −70°C or in liquid nitrogen. This system is designed to simplify the storage and retrieval of bacterial cultures.

### 3.3. Serial Dilution of Bacteria Strains

#### 3.3.1. Bacterial Colonies without Composite Sample

The strains from Microbank were revived on proliferating media: Columbia agar (Becton-Dickinson, USA) for* S. mutans* in 5% CO_2_-enriched conditions—GENbox CO_2_ (BioMérieux, France)—and for* L. acidophilus* in anaerobic conditions—GENbox anaer (BioMérieux, France). After 18-hour cultivation, bacterial emulsion in McFarland standard 0.5 was prepared. Serial dilutions of bacterial emulsion were made: 0.5 mL of dilution was added to 4.5 mL 0.9% NaCl resulting in 10 times diluted bacteria emulsion (10^−1^). Then dilutions were prepared by transferring 0.5 mL solution to the subsequent tubes. Next, 100 *μ*L of dilution was cultured on the growth medium and incubated for 24 h in 35°C. This procedure allowed us to calculate the number of bacterial colonies for given dilutions of bacterial strait without the influence of composite on bacteria.

#### 3.3.2. Bacterial Colonies with Composite Sample

The prepared samples of the composite material of Groups 0, I, II, and III were placed in 2.5 mL of 0.95% NaCl and incubated at 35°C for 3 days. Then, the material was removed from the eluate and serial dilutions of the bacteria strains were prepared. Into 1.8 mL of eluate, 200 *μ*L of the strain was introduced obtaining dilutions, in which fluoride ions released from composite materials were found effective against tested bacteria. Serial dilutions prepared for* S. mutans* and* L. acidophilus*, for both Flow Art and X-Flow composite materials, are presented in Tables 2 and 3.

In case of F2 composite material, the quantifiable bacteria colonies appeared in different dilutions. Serial dilutions are presented in Table 4.

### 3.4. Bacteria Incubation

Bacteria strains were incubated for 24 hours in eluates.* S. mutans* and* L. acidophilus* bacteria emulsion in saline incubated in the same conditions used in the control group. Next, the control and 100 *μ*L of bacteria dilution in eluate were cultivated on media to establish bacteria susceptibility:* S. mutans* on MH agar (Becton-Dickinson, USA) and* L. acidophilus* on composite media (90% Iso-Sensitest Agar + 10% Rogosa Agar) (OXOID, UK). The strains were incubated for 24 hours at 35°C. Next, bacterial colonies in the studied samples and the control groups were counted by calculating the number of bacteria per 1 mL of solution, according to the following formula:

(1) CFU/mL = number of colonies grown on the plate×10/dilution.

### 3.5. Statistical Analysis

Statistical analysis comprised the Shapiro-Wilk *W* tests for normality, Levene's test for the homogeneity of variances, and the Mann–Whitney *U* test (due to large dispersion of the data and heterogeneity of variances). Furthermore, in order to test the differences depending on the species of bacteria, type of composite material, the percentage concentration of calcium fluoride, and the specific distribution of CFU, zero-inflated Poisson regression with robust standard errors was used. A level of *p* < 0.05 was considered statistically significant. All statistical procedures were performed with the use of Stata®/Special Edition, release 14.1 (StataCorp LP, College Station, Texas, USA).

## 4. Results

**Figure 1:**
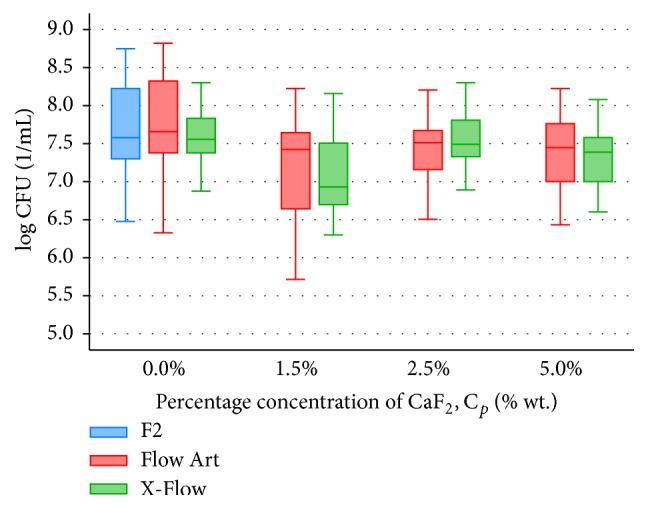
Bacteria* S. mutans* distribution in 1 mL of solution (CFU/1 mL), where composite material samples (F2, Flow Art, and X-Flow) were stored (*n* = 30).

**Figure 2 fig2:**
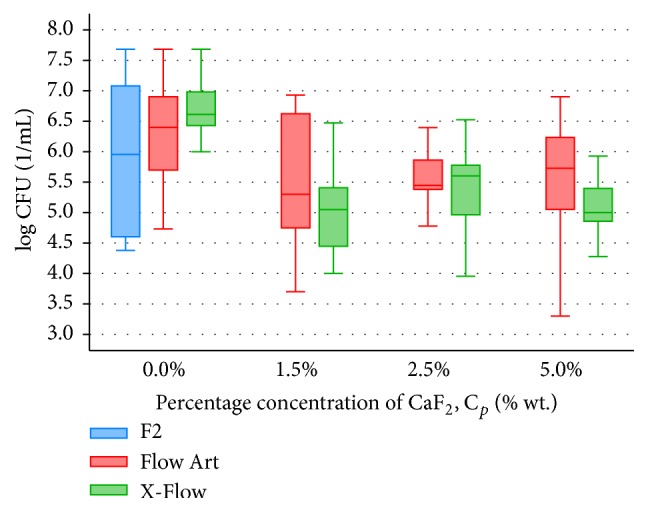
Bacteria* L. acidophilus* distribution in 1 mL of solution (CFU/1 mL), where composite material samples (F2, Flow Art, and X-Flow) were stored (*n* = 30).

**Figure 3:**
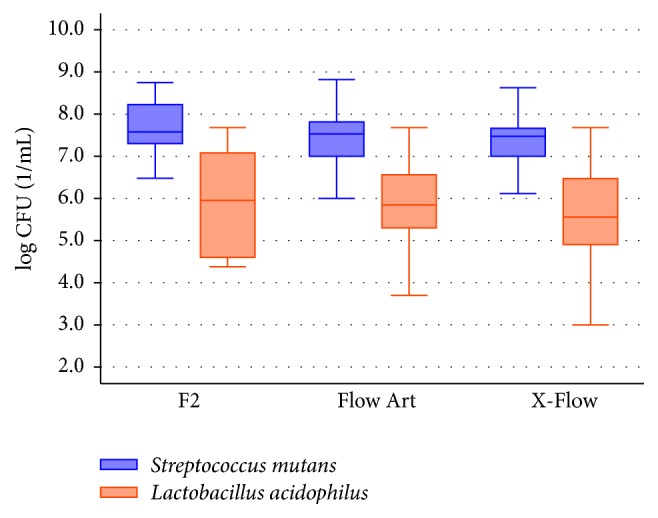
Comparison of bacteria* S. mutans* and* L. acidophilus* susceptibility/sensitivity to the composite materials, both modified and unmodified (F2: *n* = 60; Flow Art: *n* = 240; and X-Flow: *n* = 240).

After microbiological studies, bacteria cell number was counted in 1 mL of solution (CFU/1 mL). Distribution of bacteria cells for tested composite materials and bacteria were presented in Figures 1 [Fig fig2]–3.

The significantly highest (*p* = 0.025) antibacterial activity against* S. mutans*, among all tested commercially available composite materials (F2, Flow Art, and X-Flow), was shown in X-Flow (Me CFU: 3.60*∗*10^7^/mL), while the lowest was in Flow Art (Me CFU: 4.20*∗*10^7^/mL) (Figure 1).

Composite materials Flow Art and X-Flow modified with calcium fluoride showed comparable antibacterial activity against* S. mutans*; any differences were statistically insignificant (*p* = 0.396) (Figure 1). The highest antibacterial activity against* S. mutans*, for both modified composites, was observed after addition of 1.5 wt% CaF_2_ (Figure 1). Increasing the amount of CaF_2_ (over 1.5 wt%) did not influence antibacterial activity of both tested materials.

Antibacterial properties against* L. acidophilus* of three tested materials (unmodified, control groups) were comparable and statistically insignificant (*p* = 0.980) (Figure 2).

When comparing antibacterial activity of fluoride-based composite materials, Flow Art and X-Flow, against* L. acidophilus*, the statistically highest (*p* < 0.001) activity presented X-Flow containing 1.5 wt% CaF_2_ (Me CFU: 9*∗*10^4^/mL) (Figure 2). Increasing the amount of CaF_2_ (over 1.5 wt%) did not influence antibacterial activity of both tested materials.

All three tested composite materials (control and study groups altogether) show statistically greater antibacterial activity against* L. acidophilus* than against* S. mutans* (F2, *p* < 0.001; Flow Art, *p* < 0.001; and X-Flow, *p* < 0.001) (Figure 3).

## 5. Discussion

Secondary caries emerges on the filling-hard dental tissues interface. The bacteria used in the study,* S. mutans* and* L. acidophilus*, are the mainly involved in the carious process. The former one is responsible for the initiation of the process and the latter for its development [3, 7, 21, 29, 43–45].

According to many researches, 55 to 70% of fillings need replacement due to secondary caries, making it the most common reason for application of new restoration [3, 4, 10, 21, 46, 47]. The present study showed that conventional composites demonstrate little antibacterial activity against* S. mutans* and* L. acidophilus*, adversely affecting treatment outcome. Similar conclusions may be drawn based on scientific paper reviews [2, 8, 10, 48].

Bernardo et al. [2] assessed that secondary caries almost 3 times more often concerns composite material fillings than amalgams. Imazato [12] noted the increased risk of gingivitis when subgingival lesions are filled with composite materials due to their low antibacterial activity and substantial plaque deposition on their surface comparing to other restorative materials. Beyth et al. [8] and Nedeljkovic et al. [10] stated that composite materials are widely used restorative materials for their excellent esthetics and mechanical properties, but the issue of increased occurrence of the secondary caries remains unsolved. Many researchers [2, 4, 10, 48] suggest the need for development of antibacterial properties of composite materials.

For the present study, X-Flow composite material was chosen because it is very popular material with good clinical performance. While Flow Art and F2 composites exhibit also good mechanical characteristics and have similar composition, the latter contains relatively high amount of fluoride glass. Both composite materials without fluoride compounds (Flow Art) and with low fluoride content (X-Flow contains around 1% of F^−^) showed little antibacterial activity. Comparison of two unmodified materials Flow Art and X-Flow and F2 composite material revealed statistically higher antibacterial activity against* S. mutans* for X-Flow and the lowest for Flow Art. The F2 composite material that, according to manufacturer, contained fluoride glass (fluoride content is 5–7%) acts weaker than the material with no fluoride ions in composition (X-Flow). Antibacterial activities of unmodified composite materials X-Flow, Flow Art, and F2 against* L. acidophilus* were also evaluated. No statistical differences have been shown between tested materials, though all unmodified materials (X-Flow, Flow Art, and F2) presented significantly higher antibacterial activity against* L. acidophilus* than against* S. mutans*.

A number of researches [14, 26, 32, 38, 39, 49–52] proved that the highest amount of fluoride ions is released as follows: glass ionomer cements, resin modified glass ionomer cements, compomers, and the least composite materials. Authors suggest that modification of dental materials with CaF_2_-based compound could be major issue that limits dental caries development.

Calcium fluoride was used by Xu et al. [53], who incorporated CaF_2_ nanoparticles into polymer matrix of composite material. The CaF_2_ nanoparticles content was at 20%, at general content of the filler 65%. The amount of fluoride ions released from the material was approximately the same or higher than glass ionomer cements for about 2 months. Moreover, the material released phosphate and calcium ions that induced fluoroapatites formation and reduces secondary caries occurrence. The authors explained the result of their study with the size of calcium fluoride particles. Addition of nanoparticles of 1 *μ*m and smaller resulted in the substantial increase (20 times greater) in surface area of modified composite material compared to conventional composite material modified with calcium fluoride powder [26].

Galvan et al. [54] observed that releasing fluoride ions dental materials showed the highest activity on the first day after polymerization, followed by dropping for the next 90 days of the experiment remaining minimum level of F^−^ ion release. It is expected that such materials would serve as fluoride reservoirs and will remain cariostatic.

The results of the study indicated improvement in antibacterial properties of modified composite materials. Both materials, Flow Art and X-Flow, showed the highest activity when 1.5 wt% CaF_2_ was added. Comparison of CFU median for control groups of Flow Art and X-Flow with CFU median for Groups I FA and I XF against* S. mutans* showed significant improvement in antibacterial activities in groups modified with CaF_2_. Similar results were obtained for* L. acidophilus*; increasing CaF_2_ over 1.5 wt% did not decrease statistically CFU/mL number, remaining the same antibacterial activity. Groups modified with CaF_2_ possess higher antibacterial activity than F2 material containing fluoride glass.

In the present study, in order to enhance antibacterial activity of composite materials, calcium fluoride was introduced to their composition and microbiological study was performed afterwards. Most research focus on the influence of fluoride ions on* S. mutans*. Given proved cariogenic activity of* S. mutans* as well as* Lactobacillus *spp., both of those bacteria have been tested.

Statistical analysis revealed that all tested composite materials, both modified and unmodified, had statistically significant greater antibacterial activity against* L. acidophilus* than against* S. mutans* (*p* < 0.001). Naorungroj et al. [30] also compared sensitivity of* S. mutans* and* L. acidophilus* to composite materials releasing fluoride ions, but the results of their study were vague. One material showed the same level of antibacterial activity against both bacteria, while for the other two materials the activity varied. The differences in results can be explained by the differentiated resin material composition, which is not given by manufacturers in detail. Moreover, those ingredients may exhibit divers influence on bacterial cells. Naorungroj et al. [30] used enamel discs and agar diffusion method, while the present study measured influence of composite eluates on bacterial growth.

## 6. Conclusions

Within the limitations of the study the following conclusions can be made:

1. Commercially available composite materials limit bacteria growth, even higher than composite material with increased fluoride content (F2).
2. Composite materials modified with calcium fluoride reduced bacteria growth stronger than commercially available composite materials containing fluoride compounds. The greatest reduction in bacteria growth was observed for composite materials modified with 1.5% wt. CaF_2_.
3. All tested composite materials exhibit statistically greater activity against* L. acidophilus* than* S. mutans*.

## Figures and Tables

**Table 1:** Flow Art and X-Flow study groups.

Composite material	Group 0	Group I	Group II	Group III
Flow Art (FA)	Unmodified (0 FA)	+1.5% wt CaF_2_ (I FA)	+2.5% wt CaF_2_ (II FA)	+5% wt CaF_2_ (III FA)
X-Flow (XF)	Unmodified (0 XF)	+1.5% wt CaF_2_ (I XF)	+2.5% wt CaF_2_ (II XF)	+5% wt CaF_2_ (III XF)

**Table 2:** Serial dilutions for* S. mutans*.

Control group	Group I	Group II	Group III
10^−3^–10^−6^	10^−2^–10^−5^	10^−2^–10^−5^	10^−2^–10^−5^

**Table 3:** Serial dilutions for* L. acidophilus*.

Control group	Group I	Group II	Group III
10^−1^–10^−4^	10^−0^–10^−3^	10^−0^–10^−3^	10^−0^–10^−3^

**Table 4:** Serial dilutions for F2 composite material.

*S. mutans *	*L. acidophilus *
10^−3^–10^−5^	10^−1^–10^−4^
